# Diagnostic value of urinary microprotein concentration for patients with negative urinary protein test results and positive urinary casts on microscopic examination

**DOI:** 10.1002/jcla.23487

**Published:** 2020-07-19

**Authors:** ChunSheng Zheng, WenHua Wang, RongYan Chen, JiLai Liu, YangYu Li, XueJun Qin

**Affiliations:** ^1^ Department of Laboratory Medicine The Affiliated People's Hospital of Fujian University of Traditional Chinese Medicine Fuzhou China; ^2^ Department of Laboratory Medicine The First Affiliated Hospital of Fujian Medical University Fuzhou China

**Keywords:** cast, negative protein urine, urinary microprotein

## Abstract

**Objective:**

To analyze the association between positive urinary casts on microscopic examination and urinary microprotein concentration in the case of negative urinary protein test results. This study also investigated the diagnostic value of urinary microprotein examination.

**Subjects:**

A total of 949 samples that were analyzed with a UF‐1000i Urine Analyzer and returned cast alarm results were categorized into two groups, a positive and negative group, according to qualitative urinary protein sulfosalicylic acid test results. Then, 54 samples with negative protein test results but positive cast results according to microscopic examination were selected as the study group; 60 normal people with healthy physical examination results were selected as the control group. Both groups underwent urinary microprotein tests, including urinary microalbumin (mAlb), α1‐microglobulin (A1M), transferrin (TRU), and immunoglobulin G (IgG). *T* tests were used to evaluate mean differences between groups and chi‐square tests were used to calculate ratio differences between groups.

**Results:**

(a) Microscopic examinations of the positive and negative protein groups revealed no statistically significant difference in cast detection rate (*P* = .421). (b) Among the 54 samples in the study group, 37 were found to have abnormal casts, while in the remaining 17 samples, only hyaline casts were detected. (c) The detection levels of mAlb, A1M, and IgG in the study group were significantly higher than the control group (*P* values < .05).

**Conclusion:**

Urinary microprotein test should be included in the re‐examination rules for routine tests for patients with negative protein results and positive casts under microscopic examination.

## Graphical abstract

Our study shown microscopic examinations of the positive and negative protein groups revealed no statistically significant difference in cast detection rate, in the case of negative dry chemical urinary protein results, the diagnosis of the presence of casts has a certain value. So urinary microprotein test should be included in the reexamination rules for routine tests for patients with negative protein results and positive casts under microscopic examination. This would improve the detection rate of casts and reduce delays in appropriate treatment.

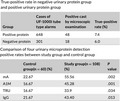

## 1 INTRODUCTION

With the increasing application of automatic urine analyzers, increased attention has been directed at improving the accuracy and efficiency of the test results. Research suggests that the results for erythrocytes, leukocytes, and epithelial cells obtained using automated analyzers are similar to the results of microscopic examinations. However, to eliminate error or uncertainty, some images (particularly those of dysmorphic cells, bacteria, yeasts, casts, and crystals) must be analyzed by manual microscopic examination.^1^ In practice, due to the limitations of the instrumental test method and the complexity of the composition of urine, a situation often arises where the chemistry result does not match the formation test result, that is, there are cases of negative protein test results with observed pathologic casts.^2^, ^3^

The presence of urinary proteins is often associated with a high risk of renal and cardiovascular diseases such as nephrotic syndrome, chronic nephritis, lupus nephritis, hypertensive nephropathy, diabetic nephropathy, and so on. Traditionally, the formation of casts is closely related to the presence of urinary proteins. The presence of pathological casts, including granular cells, epithelial cells, red cells, white cells, and waxy casts in urine is a sensitive diagnostic indicator reflecting whether the kidney has suffered substantial damage; thus, the accuracy of detection of pathological casts is critical.^4^ In the case of samples negative for urinary proteins, abnormal casts are often overlooked under microscopic examination. Hence, it is necessary to develop reasonable and effective re‐examination rules to avoid miss examinations and false examinations.

Urinary microproteins include microalbumin (mAlb), α1‐microglobulin (A1M), transferrin (TRU), and immunoglobulin G (IgG). These have important diagnostic value in diseases such as diabetic nephropathy, cardiovascular disease caused by rheumatoid arthritis, and hypertensive renal injury.^5^, ^6^, ^7^, ^8^ However, testing for these proteins is costly and time‐consuming. Immunonephelometry for urinary microproteins is particularly costly.

To date, there are no published studies on the microprotein detection rate of urinary tube type analyzers. This study aimed to analyze the relationship between casts under microscopic examination and microprotein under the condition of negative protein urine test results. Further, this study evaluated the feasibility of incorporating urinary microprotein test results into the re‐examination rules for routine urinary tests, thereby increasing the cast detection rate. The results are reported below.

## 2 SUBJECTS AND METHODS

### 2.1 Instruments and reagents

Sysmex UF‐1000i automatic urine component analyzer is a flow cytometry based for detect the urine sediment (Sysmex Corporation), matching reagents of Kairui (Kairui Corporation), and UTC‐900A quality control materials of Sysmex (Sysmex Corporation). Urinary protein were detected by a Siemens Clinitek Advantus urine analyzer (Siemens Corporation) based on PH indicator protein error method (70‐100 mg/L), matching reagents of Siemens (Siemens Corporation) and corresponding quality control materials (Siemens Corporation); an Olympus microscope is used to observe various components in urine (Olympus Corporation); Beckman Coulter IMMAGE 800 specific protein analyzer is based on a immunonephelometric method to detect the urinary microprotein, matching reagents, and Bio‐Rad quality control materials (Bio‐Rad Corporation), and sulfosalicylic acid reagent with a reagent concentration of 200 g/L.

### 2.2 Measurement of samples

Fresh morning urine samples were obtained from people hospitalized at the People's Hospital of Fujian University of Traditional Chinese Medicine from September 2017 to May 2018. A UF1000i automatic urine component analyzer and a Siemens automatic urine analyzer were used to perform urinary protein and urinary cast tests based on standardized procedures; urinary cast tests were conducted within 2 hours and urinary microprotein tests were conducted within four hours. In total, 949 samples with detected casts according to the urine analyzer were divided into two groups, a positive group and negative group, according to the results of qualitative urinary protein tests. All urinary protein samples were validated by sulfosalicylic acid. Samples in both groups were subject to centrifugal microscopic examinations performed according to standard procedures, and the true‐positive cast rates were determined for each group. In total, 54 samples negative for urinary proteins (validated by sulfosalicylic acid) were selected as the study group; 60 normal people with healthy physical examination results who were patients at our hospital during the same period of time were selected as the control group. mAlb, A1M, TRU, and IgG were tested using an IMMAGE 800 specific protein analyzer.

### 2.3 Indicators and their determination criteria

#### 2.3.1 Tube type determination criteria

The urinary cast determination criteria of the Sysmex UF‐1000i analyzer are 0‐2/μL. Microscopic examination is judged as positive for casts if the cast exceeds 1/full piece. Positive casts include granular, epithelial, red cell, white cell, and waxy casts. Criteria in practice are used for the screening test.^9^

#### 2.3.2 Urinary microprotein reference range

The reference ranges for the various urinary microproteins are as follows: urinary mAlb, 0‐19 mg/L; urinary TRU, 0‐2 mg/L; urinary IgG, 0‐8 mg/L; and urinary A1M, 0‐12.5 mg/L. Criteria were carried out according to Beckman Coulter software guide.

### 2.4 Statistical methods

The research data were processed and analyzed using SPSS 22.0 statistical software. Measurement data are expressed as means ± standard deviations ($\bar{x}$ ± *S*); *t* tests were used to compare between groups. Count data are expressed as rates (%); chi‐square tests were used to calculate differences between groups. *P* < .05 indicates a statistically significant difference.

## 3 RESULTS

### 3.1 The true‐positive rate of the negative urinary protein group and the positive urinary protein group

In total, 31.72% (301/949) of samples had a positive protein result and a negative cast result from the urine analyzer. Among the 648 samples with a positive urinary protein result and a positive cast result from the urine analyzer, 48 cases were found to have abnormal casts under microscopic examination. Among the 301 samples that had negative protein results from the urine analyzer (confirmed by sulfosalicylic acid), 18 cases were true positive as determined by microscopic examination. The results are shown in Table 1.

**TABLE 1:** True‐positive rate in negative urinary protein group and positive urinary protein group

Group	Cases of UF‐1000i tube type alarms	Positive cast by microscopic examination	True‐positive rate (%)
Positive protein	648	48	7.4
Negative protein	301	18	6.0

### 3.2 Analysis of casts in the study group

Among the 54 samples in the study group, 37 (68.52%) were found to have pathological casts; the remaining 17 (31.48%) only had hyaline casts.

### 3.3 Analysis of clinical data in the study group

**FIGURE 1:**
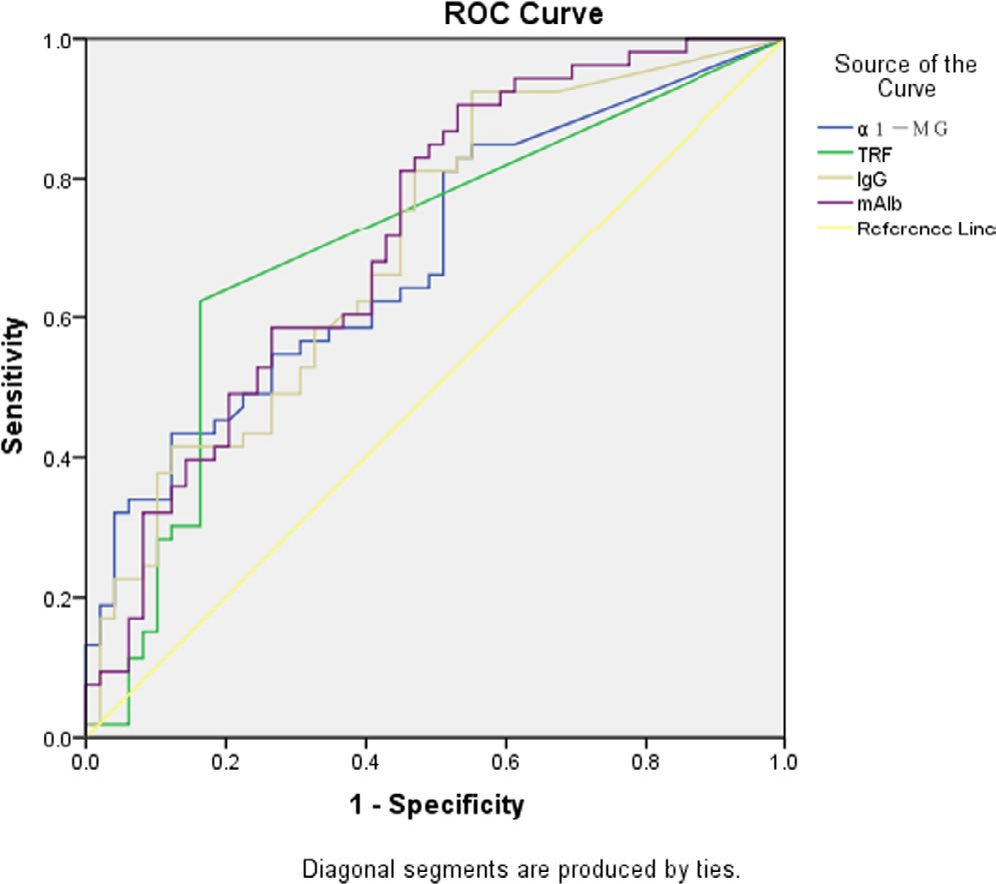
ROC curve of four urinary microproteins in the study group

In the study group, 13 patients were diagnosed with kidney disease or disease that may cause secondary renal disease, such as diabetes and hypertension. Among them, 11 cases were found to have pathological casts and two cases were found to have hyaline casts. Further, among the study group, there were 41 other types of diseases, among which 26 cases had pathological casts and 15 cases had hyaline casts (Figure 1).

### 3.4 Comparison of the positive detection rates of the four urinary microproteins between the study group and control group

The positive rates of mAlb, A1M, TRU, and IgG of two group were shown in Table 2, respectively. The positive rates of the four urinary microproteins in the study group were significantly higher than those in the control group (*P* < .05).

**TABLE 2:** Comparison of four urinary microprotein detection positive rates between study group and control group

	Control group(n = 60) (%)	Study group(n = 108) (%)	*P* value
mA	22.67	55.56	.002
A1M	16.67	45.28	.001
TRU	16.67	33.9	.034
IgG	21.67	43.40	.013

### 3.5 Comparison of detection levels of the four urinary microproteins between the study group and control group

The detection levels of mAlb, A1M, TRU, and IgG of two group were shown in Table 3. The results indicated the detection levels of mAlb, A1M, and IgG in the study group were significantly higher than the control group (all *P* < .05). The TRU level of the study group was slightly higher than that of the control group, but this difference was not statistically significant (*P* > .05).

**TABLE 3:** Comparison of four urinary microprotein test results between study group and control group (mg/L, $\bar{x}$ ± *s*)

	Control group (n = 60)	Study group (n = 54)	*P* value
mA	20.15 ± 33.49	57.95 ± 69.20	.002
A1M	2.59 ± 1.97	3.36 ± 2.79	.182
TRU	8.67 ± 7.73	21.77 ± 24.83	.000
IGU	6.84 ± 9.26	12.67 ± 13.68	.014

### 3.6 Analysis of the diagnostic value of the four urinary microproteins in the study group

According to ROC curve analysis, the areas under the curve (AUCs) for mAlb, A1M, TRU, and IgG were all >0.5, indicating statistical significant. In the case of AUC > 0.5, the AUCs of mAlb, A1M, TRU, and IgG were all in the range of 0.6‐0.9, indicating that the four urinary microproteins have diagnostic value for the detection of pathological casts.

## 4 DISCUSSION

Pathological casts are a very important part of urine sediment examinations and are the main indicator for determining whether there is pathological parenchymal damage of the kidney.^10^ The traditional view is that the presence of casts in the urine is usually accompanied by positive protein test results. However, in practical daily work, it is uncommon for a urine analyzer to report abnormal cast with negative urinary protein results. In this study, we found that the rate of negative protein results by dry chemistry with the UF‐1000i analyzer reached 31.71%, which is close to the rate published in a recent study (20.29%).^11^ Our study indicated that the true‐positive rates of the negative protein group and the positive protein group were no significant difference, indicating that there is no association between the appearance of casts in urine and whether urinary protein test results are positive. The results indicate that negative urinary protein test results with positive cast results need to be microscopically reviewed to avoid misdiagnosis. In the study group, 37 of the 54 samples were found to have pathological casts while the other 17 samples only had hyaline casts. The detection of pathological casts indicates that the kidney has suffered parenchymal injury. If casts are overlooked in negative urine protein samples, there can be severe consequences.

Next, the analysis of clinical diagnostic data in the study group revealed that 13 patients had a diagnosis of kidney disease or other disease that can cause secondary renal disease, such as diabetes and hypertension. Among them, 11 cases were found to have pathological casts. Further, 41 cases in the study group had other types of diseases, among which 26 cases had pathological casts and 15 cases had hyaline casts. There is a close relationship between the patient's clinical diagnosis and the frequency of occurrence of various casts.^12^ The relationship between hyaline casts, granular casts, and kidney disease has been reported in previous studies.^13^ The detection of negative urinary protein results together with the presence of casts has important clinical value, especially for patients who have been diagnosed with kidney disease and who may have secondary renal diseases.

Urine samples that return positive urinary protein results and negative cast qualitative test results may arise for any of the following reasons:

1. Because urinary protein detection uses an indicator error method, when the urine pH value is <3.0, urinary protein cannot be easily detected.
2. When the patient is taking a large quantity of drugs such as sulfonamides, gentamicin, penicillin, and furans, the urinary protein test may produce a false negative result.
3. Urinary protein analyzers that use dry chemistry are mainly used to detect albumin. For casts with T‐H protein as the main structure, there is a certain rate of misdiagnosis. Further, qualitative urinary protein tests mainly reflect glomerular injury, which is not sensitive to renal tubular proteinuria; qualitative protein tests are usually negative when renal tubular proteinuria occurs. Thus, routine urine examination will neglect the diagnosis of renal tubular injury.^14^ Urinary microproteins increase in early kidney damage. The detection of microproteins has several advantages, including easy material extraction, convenient and safe detection, and good sensitivity and specificity. Urinary microproteins are increasingly assessed in clinical trials to determine the development of kidney disease. The aim of doing this is to provide clinical intervention in the early stages of kidney damage to prevent irreversible kidney damage and improve the quality of life of patients.

In this study, the AUCs for mAlb, A1M, TRU, and IgG were all >0.5, indicating statistical significance (*P* < .05). In the case of negative dry chemical urinary protein results, the diagnosis of the presence of casts has a certain value. When an auto‐analyzer returns a negative urinary protein result with a cast alarm, the sample could be microscopically examined according to whether the patient's urinary microprotein level is elevated. This would lead to improvement in the cast detection rate and would avoid missed detection of casts.

The diagnostic value of the four urinary microproteins in early kidney injury has been confirmed. In particular, a number of studies have highlighted the diagnostic significance of urinary mAlb for kidney disease.^5^, ^6^, ^7^, ^8^ Studies have shown that A1M is a very good diagnostic indicator for renal tubular function damage.^15^, ^16^ Further, elevated urinary TRU may reflect early glomerular damage and decreased glomerular filtration.^17^, ^18^ Urinary IgG is also an important marker protein for early damage to the glomerular filtration membrane selective barrier and is positively correlated with glomerular damage.^19^

In the current study, we also found that the positive rate of urinary microproteins mAlb, A1M, TRU, and IgG in the study group was significantly higher than that of the control group (*P* < .05). This indicates that the levels of these four urinary microproteins in patients with negative chemical urinary protein results and positive cast results were higher than those in healthy subjects. The above results suggest that a patient's kidneys may be damaged in the case of negative urinary protein results with positive cast results. Increased urine albumin content can directly show kidney damage. A1M and IgG are important markers for the early diagnosis of renal tubular and glomerular injury and are sensitive indicators for the diagnosis of renal diseases. The detection rate and content of A1M and IgG in the study group indicated that renal tubules and glomeruli were both damaged. However, the TRU level of the study group was only slightly higher than that of the control group, and this difference was not statistically significant.

In summary, negative urinary protein cannot be used as an exclusionary standard for the presence of casts. The results of this study revealed that the cast detection rate can reach 6.0% in the case of negative urinary protein results, and the pathological cast rate accounted for 68.52% of the detected cast rate. Without microscopic examination of these samples, there will be a delay of suitable treatment. The four urinary microproteins have a certain diagnostic value for the detection of abnormal casts in the case of negative chemical urinary protein results. Hence, testing for mAlb, A1M, TRU, and IgG should be included in the re‐examination rules for routine urine tests. This would improve the detection rate of casts and reduce delays in appropriate treatment.

Based on the current results, we can conclude the following:

1. Regardless of whether the qualitative urinary protein result is positive or negative, a UF‐1000i urine analyzer cast alarm must be reexamined microscopically.
2. Urinary microprotein test results should be included in the re‐examination rules for routine urine tests; this would improve the cast detection rate and would allow prediction of whether parenchymal damage to the kidney has occurred in the early stage. Additional recheck rules can be identified and amended manually to obtain accurate and reliable results.
3. The detection of microalbuminuria can provide a comprehensive auxiliary index for the diagnosis and localization of renal injury in patients with negative urinary protein results and positive cast results; this can help meet clinical needs.

Laboratories should develop re‐examination rules that are applicable to their own circumstances and should continue to perform clinical validation and adjustment of these rules. This will greatly improve the accuracy of the test results as well as work efficiency.
